# Probing the Relationship Between the Human Gut Microbiome and Prospects of Prostate Cancer: A Systematic Review

**DOI:** 10.7759/cureus.43892

**Published:** 2023-08-21

**Authors:** Vijaya Krishna Makkena, Arturo P Jaramillo, Babatope L Awosusi, Javaria Ayyub, Karan Nareshbha Dabhi, Namra V Gohil, Nida Tanveer, Sally Hussein, Shravya Pingili, Safeera Khan

**Affiliations:** 1 Department of Internal Medicine, California Institute of Behavioral Neurosciences and Psychology, Fairfield, USA; 2 Department of Medicine, Osmania Medical College, Hyderabad, IND; 3 Department of Internal Medicine, Universidad Estatal de Guayaquil, Machala, ECU; 4 Department of Pathology and Laboratory Medicine, California Institute of Behavioral Neurosciences and Psychology, Fairfield, USA; 5 Department of Internal Medicine, Medical College Baroda, Vadodara, IND; 6 Department of Medicine, Kakatiya Medical College, Hyderabad, IND

**Keywords:** microbiome, prostate cancer, prostate neoplasia, gastrointestinal tract, microbiota, tumour of prostate

## Abstract

Prostate neoplasia is one of the most commonly occurring neoplasias in males and has a high mortality rate. Prostate cancer (PCA) risk factors include tall stature, male sex, known family history, obesity, high blood pressure, lack of fitness, higher levels of testosterone for a long time, increasing age, and ethnicity are well known. The association and role of the gut microbiota in different diseases in our body have been highlighted recently. Therefore, finding the influence of gut microbiota on the prostatic cells can be useful for preventing prostatic neoplasia and/or reducing its severity. We aimed to assess its impact on PCA risk. We thoroughly searched databases for the relevant literature for our systematic review. The final research papers analyzed how bacteria played a role in the risk of PCA, either through inflammation or the production of metabolites that increase/decrease the risk of PCA. Based on the studies reviewed, we found that some gut bacteria play a role in the formation of PCA. In contrast, some bacteria can help prevent PCA, but the metabolism of the dietary components is the major factor for PCA.

## Introduction and background

Carcinoma of the prostate is the most common male cancer diagnosed worldwide, with mortality ranking fifth in men, roughly 1.5 million newly discovered cases, and 0.375 million annual deaths worldwide in 2020 [1]. It is also the most often diagnosed cancer worldwide in more than 50% of nations (112 of 185) [2,3]. In the United States, it is second in cancer deaths and first in most frequently diagnosed organ cancer among males [2]. Male gender, advanced age, BMI over 30, elevated blood pressure, increased height, a lack of activity, known family history of prostate neoplasia, long-term elevation of androgens (testosterone), and race are all risk factors for prostate cancer (PCA). Fortunately, cancer aggressiveness declines with age, even if the incidence rises as people age [4].

For the prostate to function properly, testosterone is very much needed; thus, testosterone deprivation therapy is used to treat PCA, which is an effective modality. Normally, starting with the peripheral basal cells, cancer develops when normal prostate glandular cells undergo mutation. The most common place where the neoplasm arises is the prostate's posterior zone, which can be palpated by digital rectal examination for identification of cancer. Adenocarcinoma, known as PCA, shows typical glands on microscopy because it arises from that part of the prostate. A tumor nodule is created as a result of the cancer cells' growth and multiplication, which originally spread to the nearby. It can grow out of a prostate capsule or locally inside the prostate for many years. Bones and lymph nodes are frequent sites of metastasis for PCA. It is believed that the venous plexus at the prostate can allow the metastasis to spread to the bones.

The cancer is either treated by prostatectomy or hormonal therapy/chemotherapy/radiotherapy, depending on the cancer stage. Patients with hormone-sensitive PCA with metastasis had longer survival times when receiving androgen deprivation therapy (ADT) in addition to abiraterone acetate or docetaxel chemotherapy [5]. This clinical study has produced important results showing that adding abiraterone acetate to ADT significantly positively impacted patients in the early stages of the disease [6]. The androgen resistance continues to be the primary motivator in most castration-resistant PCA (CRPC) patients. Late-stage clinical research is being conducted on new second-generation androgen receptor (AR) antagonists that have increased effectiveness in overcoming resistance mechanisms and fewer adverse effects [7]. The emergence of androgen-independent (castrate-resistant) PCA is connected to the activity of different protein kinases.

Protein kinases have a role in developing aggressiveness and spreading prostate neoplasia. Certain protein kinase inhibitor treatments are useful here because some of them are implicated in the signaling pathway of ARs with the potential to alter the cellular response to androgen deprivation. There are a lot of research possibilities in the field of protein kinase inhibitors that would block kinase-mediated signal pathways or lower particular kinase activity [8]. Clinical studies on metastatic CRPC have shown that the poly (ADP-ribose) polymerase (PARP) inhibitor (niraparib) is efficient and secure. Similar PARP inhibitor medications, talazoparib, and ipatasertib, are also being tested in clinical trials [9]. Chimeric antigen receptors, bispecific T-cell engager immunological therapies, immune checkpoint inhibitor combos, and other novel immunotherapy medicines are being developed, and preliminary test findings are encouraging [10]. Personalized markers on PCA cells can be targeted with radiopharmaceuticals like lutetium 177 to provide targeted, tailored therapy. One of the earliest therapies based on this therapeutic method is lutetium [11].

By providing lethal material to malignant cells, targeted, specifically designed anticancer medicines have great promise for decreasing side effects and managing malignancy. Using designed ankyrin repeat proteins (DARP) is one technique. The fatal payload of these non-immunoglobulin scaffold proteins exclusively targets PCA cells. In 40% to 60% of prostate tumors, epithelial cell adhesion molecule (EpCAM) is overexpressed. Accelerated tumor growth, a higher risk of metastasis, treatment resistance, and a loss in cancer-specific survival are all associated with it. The experimental in vitro transport of a *Pseudomonas* exotoxin A variant into EpCAM-expressing PCA cells was achieved utilizing a specifically designed DARP molecule. Normal prostatic cells were not damaged by the toxin's quick internalization [12].

All species, from cnidarians to humans, have microbial populations, but the gut is where these host-associated bacteria are most diverse and numerous. Membership in what is known as the gut microbiome can be as straightforward as one bacterial species or comprise hundreds to thousands of different germs from various life domains. The host's gut microbiome and its effects on the host can range from advantageous to harmful; interactions may depend on the setting and affect different aspects of the host's physiology and organ systems [13].

Numerous illnesses, including colon cancer, rheumatoid arthritis, and Alzheimer's, are linked to gut microbiota. An immunological function is modulated by the gut microbiota, which affects how the body reacts to immunological checkpoint treatment. According to lifestyle, nutrition, sex, ethnicity, genetics, and geography, the gut microbiota varies. Diet and lifestyle, in particular, significantly impact the onset and spread of PCA. Recent research has shown a link between PCA and the gut flora. A high-fat diet (HFD) results in gut dysbiosis and the release of bacterial metabolites, including short-chain fatty acids and phospholipids, into the bloodstream, which aids PCA growth. The gut microbiota's potential role as a testosterone source may impact PCA development. There is an increased risk of castration-resistant PCA [14].

The recent advances in the gut microbiome describe how the gut influences health by modulating inflammation in our body. In the last few decades, there has been a huge amount of research done about gut microbiota which revealed gut dysbiosis can be linked to pathologies in the human body, such as dental caries and periodontitis, many metabolic disorders, chronic bowel inflammatory conditions, cardiovascular diseases, neoplasia, chronic kidney diseases [15-21]. For a specific disease to begin, there's a need for increased permeability of the gut barrier, which can be due to any condition, but associated dysbiosis is a factor in it. As described above, many factors influence the risk of PCA, but the way the gut influences PCA is not explored in detail. Our systematic review aimed to explore the role of microbiota in the gut affecting the risk of developing PCA.

## Review

Methods

This systematic review used the Preferred Reporting Items for Systematic Reviews and Meta-Analyses (PRISMA) 2020 guidelines [22].

Search Sources and Strategy

We looked in PubMed, PubMed Central (PMC), MEDLINE, and Cochrane Library for pertinent publications. We used various combinations of keywords: gut microbiota, prostate cancer, and prostate neoplasia in different combinations to search all databases. In PubMed, however, along with these keywords, the following strategy was developed and used to search relevant literature in PubMed's MeSH database: (("Gastrointestinal Microbiome/genetics"[Majr] OR "Gastrointestinal Microbiome/physiology"[Majr] )) AND (( "Prostatic Neoplasms/diagnosis"[Majr] OR "Prostatic Neoplasms/epidemiology"[Majr] OR "Prostatic Neoplasms/etiology"[Majr] OR "Prostatic Neoplasms/genetics"[Majr] OR "Prostatic Neoplasms/physiopathology"[Majr])). Table 1 shows the databases used and the identified numbers of papers for each database.

**Table 1:** Keywords/strategy used and the number of identified papers

Keywords/search strategy	Database used	No. of papers identified
(( "Gastrointestinal Microbiome/genetics"[Majr] OR "Gastrointestinal Microbiome/physiology"[Majr] )) AND (( "Prostatic Neoplasms/diagnosis"[Majr] OR "Prostatic Neoplasms/epidemiology"[Majr] OR "Prostatic Neoplasms/etiology"[Majr] OR "Prostatic Neoplasms/genetics"[Majr] OR "Prostatic Neoplasms/physiopathology"[Majr] ))	PubMed Mesh	6
Gut microbiota and prostate cancer	PubMed	130
Gut microbiome and prostate cancer	PubMed	112
Gut microbiota and prostate cancer	MEDLINE	101
Gastrointestinal microbiome and prostate cancer	PubMed	77
Gut microbiota and prostate neoplasia	PubMed	61
Gut microbiota and prostate cancer	Cochrane Library	19

*Inclusion* *and* *Exclusion* *Criteria*

**Figure 1:**
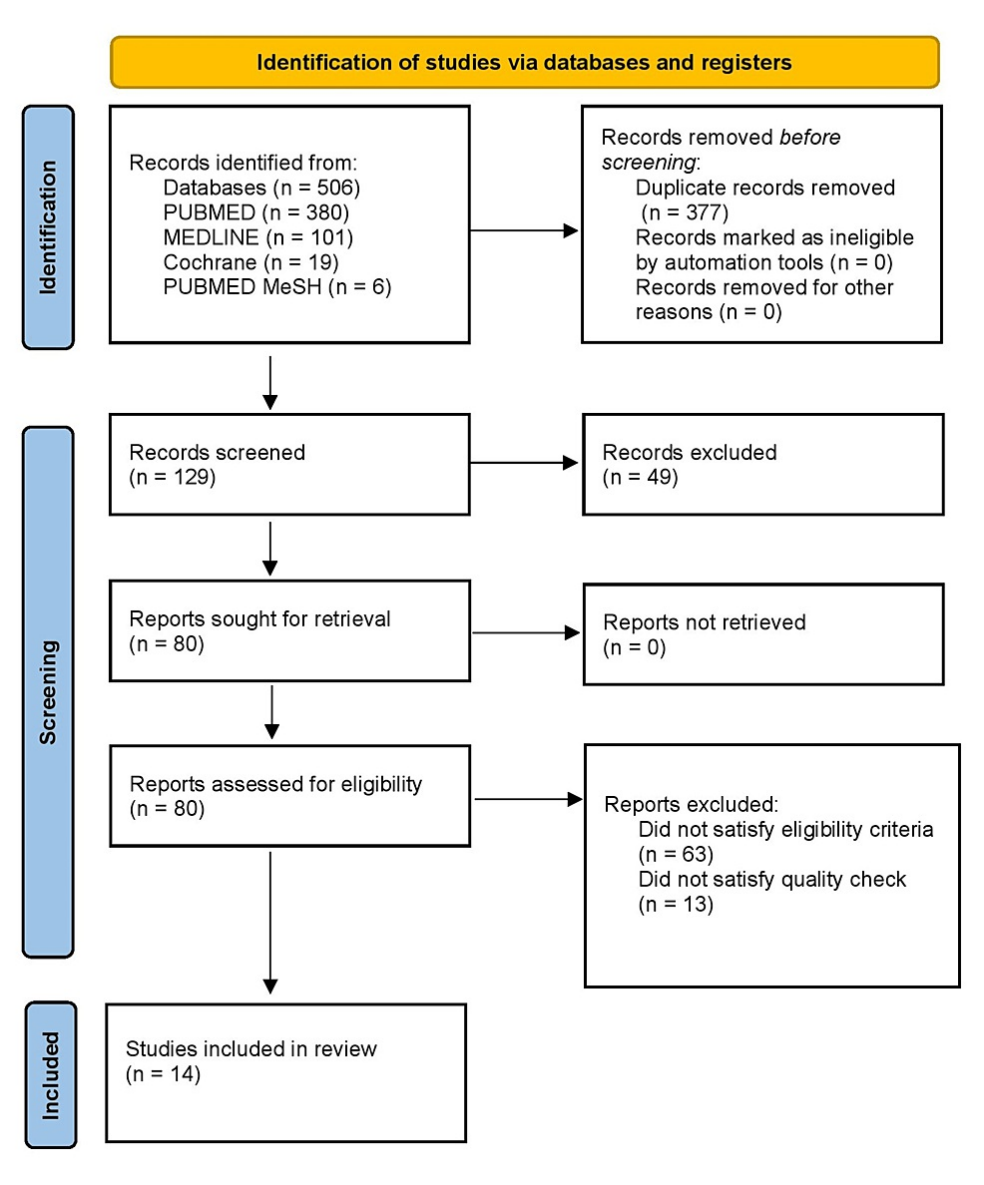
PRISMA flowchart showing the methods used to identify studies, quality check, and the finalized articles

We chose the most recent publications and journals that have been released during the last 10 years, including any papers that had been authored in English or for which there was an English full-text translation. Only research publications with mixed methodologies and people as subjects were selected. When the complete text of the papers could not be acquired, articles were disqualified. We did not include articles on how to cure PCA or the manner in which the gut affects how chemotherapy treatments work. Additionally eliminated were proposal papers and grey literature as shown in Figure 1.

The articles were assessed for eligibility using the relevant quality appraisal tools. Table 2 shows the results of the quality appraisal.

**Table 2:** Quality appraisal using their respective tools SANRA: a scale for the assessment of narrative review articles, JBI: Joanna Briggs Institute

Study	Checklist used	Quality
Crocetto et al., 2020 [23]	SANRA	8/12
Fujita et al., 2022 [14]	SANRA	9/12
Fujita et al., 2023 [24]	SANRA	10/12
Garbas et al., 2021 [25]	SANRA	9/12
Katongole et al., 2020 [26]	SANRA	9/12
Kucera et al., 2020 [27]	SANRA	8/12
Kustrimovic et al., 2023 [28]	SANRA	9/12
Massari et al., 2019 [29]	SANRA	8/12
Matsushita et al., 2021 [30]	JBI checklist	7/10
Matsushita et al., 2022 [31]	JBI checklist	7/10
Matsushita et al., 2023 [32]	SANRA	8/12
Mirzaei et al., 2021 [33]	SANRA	8/12
Reichard et al., 2022 [34]	JBI checklist	9/10
Sha et al., 2020 [35]	SANRA	9/12
Yang et al., 2023 [36]	SANRA	8/12

Outcomes Measured

The involvement of microbiota development in PCA was the main conclusion evaluated from the finished study articles. The secondary outcomes evaluated were the effects of other variables on gut bacteria, which in turn affected PCA.

Study Characteristics

Three of the 15 research publications we looked at were observational studies, while the other 12 were narrative reviews. All studies showed how the gut microbiota influences immunity and other systems through inflammation and dietary influence in our body and how this affects the prostate. A total of 15 articles were reviewed in this systematic review. Table 3 shows the types of all included studies and their results.

**Table 3:** Types of the included studies and their results PCA: prostate cancer, ROS: reactive oxygen species, SCFA: short-chain fatty acid, IL6: interleukin-6, STAT3: signal transducer and activator of the transcription-3, LPS: lipopolysaccharide, LNCaP: lymph node carcinoma of the prostate, LAPC4: Los Angeles prostate cancer 4, AR: androgen receptor

Authors and year of publication	Type of the study	Result of the study
Crocetto et al., 2020 [23]	Traditional review	PCA patients had higher amounts of *Bacteroides* spp. in their guts.
Fujita et al., 2022 [4]	Traditional review	Ruminococcus growth results in higher phospholipid levels and a higher risk of PCA.
Fujita et al., 2023 [24]	Traditional review	PCA risk is enhanced by increased *Ruminococcus*, which also causes increased androgenesis.
Garbas et al., 2021 [25]	Traditional review	Inflammation, ROS production, and prostatic cell dysplasia are all enhanced along with free estrogen levels.
Katongole et al., 2020 [26]	Traditional review	*Faecalibacterium prausnitzii* concentrations are higher in PCA patients.
Kucera et al., 2020 [27]	Traditional review	Patients with high SCFA levels had lower levels of *Enterobacteriaceae* due to dietary changes that increased their risk of PCA.
Kustrimovic et al., 2023 [28]	Traditional review	PCA risk was reduced with *Prevotella*.
Massari et al., 2019 [29]	Traditional review	In PCA patients, glucose metabolism by *Bacteroides* and streptococcus predominates, enriching pro-inflammatory symptoms.
Matsushita et al., 2021 [30]	Observational study	Patients with high-risk PCA had higher *Rikenellaceae*, *Alistipes*, and *Lachnospira* in their gut microbiota.
Matsushita et al., 2022 [31]	Observational study	Significant correlations were found between the proportion of *Firmicutes* in the gut microbiota and blood total testosterone levels.
Matsushita et al., 2023 [32]	Traditional review	The IL6-STAT3 axis is stimulated when prostatic local histamine signaling is triggered by LPS (released in the event of a leaky gut).
Mirzaei et al., 2021 [33]	Traditional review	In LNCaP and LAPC4 PCA cells, sodium butyrate decreases the expression of the AR, which plays a crucial role in developing and spreading PCA.
Reichard et al., 2022 [34]	Observational study	Aggressive PCA was more likely to develop when choline, betaine, and phenylacetylglutamine were present.
Sha et al., 2020 [35]	Traditional review	Alterations in intestinal permeability brought on by antibiotic-induced microbiota alterations lead to systemic inflammation and increase the risk of PCA.
Yang et al., 2023 [36]	Traditional review	A rise in *Lachnospira*, *Subdoligranulum*, *Lachnobacterium*, and Christensenellaceaewas seen in high-grade PCA.

Discussion

Until recently, there was no known connection between gut microbiota and PCA because the gut and the prostate are different organs. According to several studies, PCA development and resistance may be related to the gut microbiome.

Bacterial Association in PCA

While overall bacterial load and *Eubacterium* abundance were linked with host tumor hypermutation, *Escherichia *and *Acidovorax* were considerably abundant. Additionally, *Bacteroides massiliensis* contributes to the rising incidence of prostatic cancer, and a study shows increased levels of *Bacteroides* spp. in high-grade PCA [23,25]. *Subdoligranulum*, *Lachnobacterium*, Christensenellaceae, *Lachnospira*, Rikenellaceae, *Eggerthella*, and *Alistipes *are members of the SCFA-producing order clostridiales and were shown to be more prevalent in individuals with high-grade PCA. These findings imply that SCFAs could be very important in developing PCA. *Anaerofilum* is protective because it reduces inflammation caused by *Alistipes*, which produce pro-inflammatory mediators and raise the risk of PCA [30,36].

*Akkermansia muciniphila* extracellular vesicles interact with CD8-positive T-cells and macrophages to stimulate anti-tumor immunity [32]. Although the bacterial growth in American patients differed from what was observed in the current investigation on Japanese patients, there were parallels in the metabolic activities of the Japanese and American cohorts. This implies that certain bacterial metabolites, not individual bacteria, are responsible for PCA worldwide [30]. The hyperandrogenic conditions, the medications taken by PCA patients, or the food, which promotes bacterial growth but is not the only indicator of the risk for PCA by gut microbiota, can all contribute to this elevated bacterial population.

Pathogenesis of PCA

Insulin-like growth factor (IGF-1), mostly produced by the liver and muscles, is crucial for the growth of bones and muscles. Additionally, PCA cells release IGF-1 in an autocrine manner, which promotes PCA development by activating the mitogen-activated protein kinases (MAPK) and phosphoinositide 3-kinases (PI3K) signaling pathways. Through both systemic and local prostate IGF-1, SCFAs, a significant gut microbiota metabolite, control PCA development. *Ruminococcus* can convert pregnenolone and hydroxypregnenolone to dehydroepiandrosterone (DHEA) and testosterone, which are downstream metabolites. Abiraterone acetate, a specific inhibitor of CYP17A1, prevents the bacterial conversion of pregnenolone to DHEA and testosterone. Interestingly, *Ruminococcus* possesses genes with significant sequence similarity with human CYP17 [14]. Bacterial substances such as lipopolysaccharides (LPS) and lipoteichoic acids (LTA) can enter the systemic circulation due to a leaky gut. Leaked LPS and LTA then cause systemic inflammation, which has a variety of impacts, including ones that can cause cancer. Due to increased gut permeability brought on by antibiotic-induced dysbiosis, defined by the abundance of Proteobacteria, tumor LPS was elevated. PCA growth was facilitated by the nuclear factor-beta (NF-B)-IL6-STAT3 axis being triggered by an intratumoral increase of LPS [24].

*Bacteroides massiliensis* also contributes to the increased occurrence of PCA by raising free estrogens in the circulation as a result of glucuronidases. Increased reactive oxygen and nitrogen species and increased free estrogens result in apurinic DNA sites that trigger mutations and the beginning of oncogenesis in prostate cells [25]. However, the LPS/toll-like receptor-4 (TLR4) signaling block inhibited HFD-induced PCA growth. By enhancing myeloid-derived suppressor cells and activating the IL-6-STAT3 axis, prostate local histamine signaling induced by LPS can accelerate the formation of inflammatory PCA [32].

The hormone testosterone significantly regulates male health, and aging-related declines in testosterone levels (late-onset hypogonadism) are strongly linked to several health issues, such as metabolic syndrome, muscular weakness, and sexual dysfunction. In addition, testosterone contributes to benign prostatic hyperplasia and PCA. The number of *Firmicutes* and serum testosterone levels were positively associated with older male participants. We found a significant correlation between the abundance of *Firmicutes* and blood testosterone levels without regard to age, BMI, or lipoprotein levels. In Japanese men, *Firmicutes* had a higher impact on increased testosterone than low testosterone, even though age, BMI, and lipoprotein levels have been demonstrated to affect testosterone levels [31].

Diet and Gut Microbiota and PCA

Burkina Faso children had considerably lower *Enterobacteriaceae* levels in their gut microbiota (P<0.05) and significantly higher levels of short-chain fatty acids (P<0.001) in their feces. In the study, children from Burkina Faso, who have a high-fiber diet, were compared to children from Europe, who consume a conventional Western diet [27]. Although the gut microbiota does not directly affect the prostate, it may do so through the actions of cytokines, immune cells, or bacterial metabolites and components that are absorbed from the intestine and circulate throughout the body (referred to as a "microbiota-gut-prostate axis"). These findings suggest that diet and nutrition may affect PCA, with gut flora as a partly mediating factor. According to our predictions, the "microbiota-gut-prostate axis" may become more well-understood in the future [7].

The most common SCFA in the colon is butyric acid, produced by the breakdown of acetic acid by *Faecalibacterium prausnitzii*. Butyric acid exerts anti-tumor actions through the induction of apoptosis and proliferation restriction. Notably, HFD results in a leaky gut, which makes it possible for various metabolites and bacterial fragments to enter the host's systemic circulation and induce illnesses like endotoxemia. Thus, this event can potentially control the inflammatory response and affect how PCA development is controlled. Additionally, *Prevotella stercorea* reduced the synthesis of testosterone and the risk of PCA [7].

In LNCaP and LAPC4 PCA cells, sodium butyrate decreases the expression of the AR, which plays a crucial role in developing and spreading PCA. However, current research suggests that SCFAs may contribute to PCA through IGF-1. These lipids are often required in malignancies with high cell turnover when acetate is present [14]. The use of propionates decreases the probability of human PCA spreading. The intestinal flora processes these three metabolites of a high-carbohydrate meal [33]. Men who have greater levels of choline, betaine, or PCA-related metabolites linked to the gut microbiome (such as phenylacetylglutamine) have around two times as many chances (compared to Q1) of developing incident PCA cancer in the future.

PCA patients also have dysregulated choline metabolism, especially when choline kinase, an enzyme that helps with the rate-limiting step in phosphatidylcholine production, is overexpressed [34]. Another study found that antibiotics alter the local gut microbiota, which alters intestinal permeability, causes systemic inflammation, and raises the risk of PCA by activating the STAT3 and IL-6 axis [35].

Limitations

Despite a thorough search encompassing several databases, no clinical trials examining the influence of gut microbiota on the prostate were identified. The majority of the published literature consisted of narrative reviews and observational investigations.

## Conclusions

This research paper analyzed the gut microbiome's role in PCA risk. Based on the studies reviewed, we found that some gut bacteria play a role in the formation of PCA. In contrast, some bacteria play a role in preventing the formation of PCA, but the metabolism of the dietary components is the major factor for PCA. Another possible mechanism is the bacteria's excessive testosterone production, which leads to prostatic tissue proliferation and dysplasia. The gut modulation by antibiotics also leads to the formation of a leaky gut, which leads to systemic inflammation causing mutation in prostatic tissue leading to PCA.

The emphasis is upon the gut microbiome, as bacteria in the gut have been in for a long time in our body, and their modulation can present various problems, one of which is PCA. In the future, other mechanisms should be elicited upon how the specific bacterial growth inhibition/promotion has a role in the formation/prevention of PCA and how putting transplanting the bacteria into the gut can be helpful in risk reduction, and more clinical studies need to be done regarding it.
